# A Kinodynamic Model for Dubins-Based Trajectory Planning in Precision Oyster Harvesting

**DOI:** 10.3390/s25154650

**Published:** 2025-07-27

**Authors:** Weiyu Chen, Chiao-Yi Wang, Kaustubh Joshi, Alan Williams, Anjana Hevaganinge, Xiaomin Lin, Sandip Sharan Senthil Kumar, Allen Pattillo, Miao Yu, Nikhil Chopra, Matthew W. Gray, Yang Tao

**Affiliations:** 1 Fischell Department of Bioengineering, University of Maryland, College Park, MD 20742, USA; wchen137@umd.edu (W.C.); cyiwang@umd.edu (C.-Y.W.); ahevagan@umd.edu (A.H.); 2 Department of Mechanical Engineering, University of Maryland, College Park, MD 20742, USA; kjoshi@umd.edu (K.J.); mmyu@umd.edu (M.Y.); nchopra@umd.edu (N.C.); 3 Horn Point Laboratory, University of Maryland Center for Environmental Science, Cambridge, MD 21613, USA; awilliams@umces.edu (A.W.); mgray@umces.edu (M.W.G.); 4 Department of Electrical Engineering, University of South Florida, Tampa, FL 33620, USA; xlin2@usf.edu; 5 Maryland Applied Graduate Engineering, University of Maryland, College Park, MD 20742, USA; sandip26@umd.edu; 6 University of Maryland Extension, College of Agriculture and Natural Resources, University of Maryland, College Park, MD 20742, USA; dapatt@umd.edu

**Keywords:** boat maneuvering, precision aquaculture, kinodynamic model, motion primitives, trajectory following

## Abstract

**Highlights:**

**What are the main findings?**

- We developed a novel hybrid kinodynamic model combining the Dubins and Nomoto models to map steering input directly to spatial coordinates for underactuated boats.
- Field experiments in oyster aquaculture environments showed turning radius errors within 1.5% and trajectory following accuracy with sub-meter precision across various path complexities.

**What are the implications of the main findings?**

- Enable efficient and precise motion planning for autonomous oyster harvesting vessels under real-world constraints like starboard-turn-only paths.
- Provide a scalable foundation for integrating control systems and MIMO-compatible models in future aquaculture automation frameworks.

**Abstract:**

Oyster aquaculture in the U.S. faces severe inefficiencies due to the absence of precise path planning tools, resulting in environmental degradation and resource waste. Current dredging techniques lack trajectory planning, often leading to redundant seabed disturbance and suboptimal shell distribution. Existing vessel models—such as the Nomoto or Dubins models—are not designed to map steering inputs directly to spatial coordinates, presenting a research gap in maneuver planning for underactuated boats. This research fills that gap by introducing a novel hybrid vessel kinetics model that integrates the Nomoto model with Dubins motion primitives. Our approach links steering inputs directly to the vessel motion, enabling Cartesian coordinate path generation without relying on intermediate variables like yaw velocity. Field trials in the Chesapeake Bay demonstrate consistent trajectory following performance across varied path complexities, with average offsets of 0.01 m, 1.35 m, and 0.42 m. This work represents a scalable, efficient step toward real-time, constraint-aware automation in oyster harvesting, with broader implications for sustainable aquaculture operations.

## 1. Introduction

Although the United Nations Food and Agriculture Organization (FAO) has ranked the US highly for potential aquaculture industry growth due to the large available areas in bays [1], the current domestic production remains hindered by outdated practices. This is especially true in on-bottom oyster farming, where cultivation and harvesting methods have changed little in over 200 years.

Current dredging methods rely heavily on manual operation and lack any form of trajectory planning. Vessel captains often navigate oyster leases by circling randomly, resulting in redundant seabed disturbance, inefficiencies in shell usage, and long-term environmental degradation [2]. From a control theory perspective, this reflects a fundamental absence of models that connect steering input directly to the spatial state, making precise path execution infeasible. Existing autopilot systems and guidance strategies—designed primarily for open-water, long-distance waypoint tracking—do not meet the sub-100-m, decimeter-level precision required in aquaculture harvesting [3]. As noted by Kjerstad and Fossen [4], traditional line-of-sight guidance assumes near-constant velocity and ignores the maneuvering constraints of underactuated marine vessels.

To overcome these limitations, we introduce a hybrid kinodynamic model that maps normalized steering inputs directly to the planar position and heading. This feedforward model integrates the empirically validated second-order Nomoto model with Dubins path geometry, allowing for constraint-aware motion generation using only the input history—without relying on intermediate control states like the yaw rate or angular acceleration.

Our work draws inspiration from related advances in control-based trajectory generation. Tedrake’s work on control-affine systems for UAVs [5] demonstrated the value of mapping the control input directly to the system state in constrained environments. Majumdar [6] and Althoff [7] extended these ideas using reachability-based methods to guarantee safe navigation in ground and aerial robots. While their approaches emphasize feedback synthesis and safety margins, our method focuses on a lightweight, field-deployable feedforward model that is tailored to aquaculture operations where steering constraints (e.g., starboard-turn-only due to dredge positioning) dominate the path feasibility.

Unlike prior ship dynamics models that output yaw or yaw rate, our model generates real-world Cartesian trajectories that are suitable for generating precise operator instructions in a practical setting. This formulation enhances the control quality in field conditions by reducing operator variability and making repeatable path execution feasible—without requiring onboard feedback or dynamic re-planning. The model also supports further integration with future closed-loop systems and dredge localization modules.

**Figure 1:**
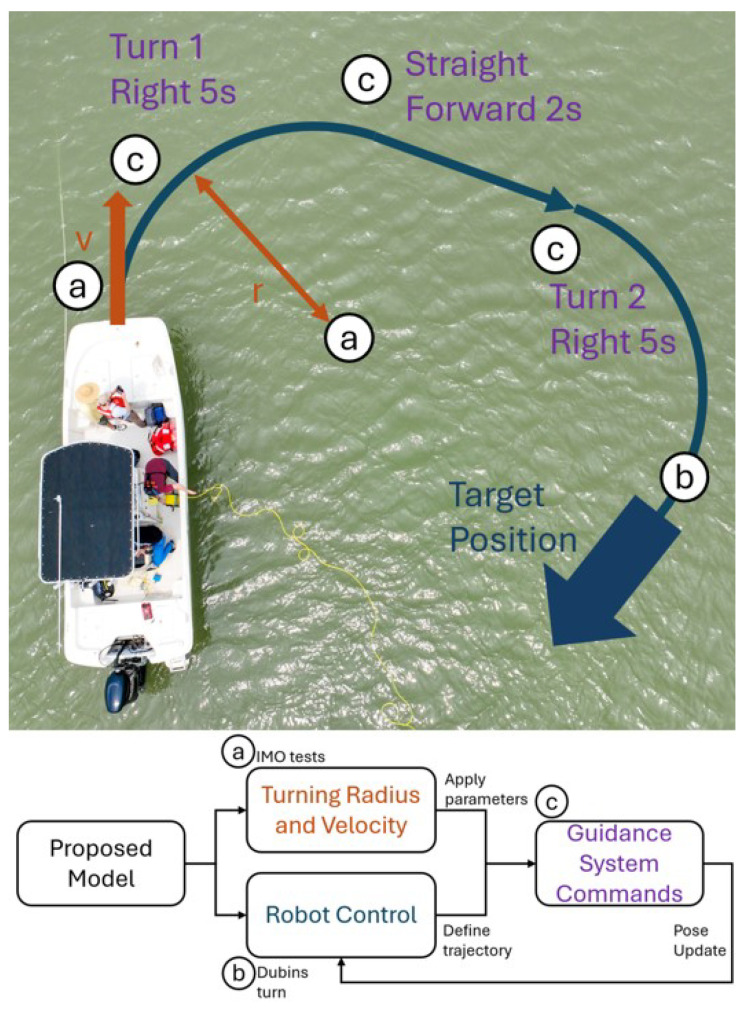
System overview of the proposed trajectory planning framework for autonomous oyster harvesting. Field tests demonstrate how the approach enables precise, constraint-aware maneuvers using only right-turning commands, consistent with dredge deployment practices in aquaculture.

To the best of the authors’ knowledge, there remains a relative lack of research focused on kinodynamic models that are specifically designed for motion planning in underactuated ships. Even fewer studies explore the application of autonomy within aquaculture, which the authors primarily attribute to the sector’s slow pace of technological advancement. In this context, no existing research has proposed a kinodynamic model that is tailored to aquaculture-related planting and harvesting operations. The development of such a model not only enables the creation of control systems for full-coverage path planning but also draws on several novel start-to-end control methods that were originally developed for aircrafts, which rely on direct mappings from user inputs to Cartesian coordinates. This type of input-to-coordinate mapping also facilitates the generation of custom driving instructions for a given trajectory in the Cartesian plane, as illustrated in Figure 1. Therefore, a kinodynamic model that links boat steering inputs to spatial coordinates—along with a framework for calculating associated driving commands—would not only enhance current coverage path planning techniques but also bridge prior work in robotic coverage with emerging needs in ship control and aquaculture automation.

Given the background mentioned above, this paper develops a kinodynamics model that directly maps steering input to the (x,y,θ) coordinates of a boat. The key contributions of this work are as follows:

- Novel integration of the Nomoto and Dubins models, specifically designed for aquaculture applications and leveraging the strengths of both models—such as easy system identification and minimum turning radius calculation.
- Direct steering-to-coordinate mapping, unlike existing models that rely on indirect approximations, improving the precision in full-coverage path planning.
- Experimental validation in a real-world oyster harvesting environment, using gray-box system identification on a Carolina Skiff Model 21 DLX.
- Demonstration of practical feasibility for precision aquaculture, with motion primitives computed from identified model parameters and tested at the Horn Point Laboratory in Cambridge, MD.

## 2. Related Work

### 2.1. The Dubins Model

**Figure 2:**
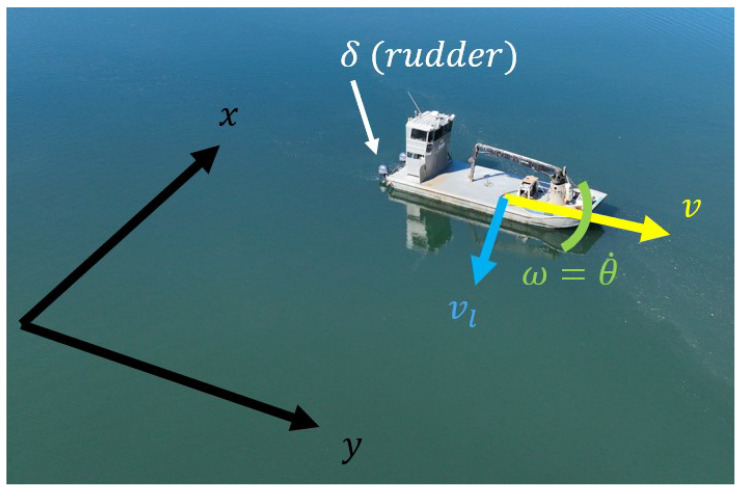
Graphical illustration of the definitions used throughout this paper. Dots above denote derivatives of a variable ($\dot{\theta }$ denotes the first derivative of the yaw).

The Dubins vehicle model [8,9] describes the shortest path for vehicles with a limited turning radius, which in most cases are underactuated vehicles. Due to its properties as a nonlinear system that maps the angular velocity ($\omega =\dot{\theta }$) to the (x,y,θ) coordinates (Notations and axis defined in Figure 2), it is widely used in path planning for vehicles with turning constraints in general [10,11,12]. In the case of coverage path planning, although the output is the same as our C space, the input being the angular velocity leads to the same problem as in the Nomoto model. Therefore, the Dubins model must also be used alongside another model to map the input to the configuration space (C space). Taking inspiration from the Dubins vehicle model, this research aims to create a model that maps the relationship between the steering input and the C space of the coverage path planning problem.

(1) $$\begin{array}{ccc} \; & \dot{\eta }=f\left(\eta ,u\right)=\left[\begin{array}{cc} \cos(\eta_{3}) & 0\\ \sin(\eta_{3}) & 0\\ 0 & 1 \end{array}\right]u,\; & u=\left[\begin{array}{c} v\\ \omega \end{array}\right] \end{array}$$

Path planning and controls for ships based on the Dubins model have also been a well-researched topic [13,14,15]. However in contrast to the research carried out on the Nomoto model, they mainly focus on the kinetics of the boat, assuming that the ship’s yaw can be a controlled input in and of itself. Therefore, this research seeks to fill a gap between the research conducted with both the Nomoto model and the Dubins model to provide a novel solution for harvesting and full-coverage path planning in aquaculture.

The major advantages that the Dubins vehicle model brings are (1) that the minimum turning radius of a vehicle is $\frac{1}{u_{max}}$, where $u_{max}$ is the maximum input available to the system. This feature of the Dubins vehicle model allows model verification to be based on the turning radius alone. (2) In addition, any planar R-geodesic in the Euclidean space consists of at most three maneuvers.

### 2.2. Kinodynamic Boat Models

In the Euclidean space, any Dubins path consists of at most three maneuvers. However, while the Dubins model is helpful in determining path shapes under turning constraints, it cannot represent the physical dynamics of boats. To address this limitation, we now review commonly used kinodynamic models that describe the actual motion behavior of marine vessels.

The motion of boats can be described using the six-degree-of-freedom (6-DOF) equations of motion, which account for the nonlinear interactions between hydrodynamic forces, inertial effects, and external inputs. These equations, widely used in maritime robotics and control applications, are expressed as Equation (2) in [3,16].

Fossen’s equations of motion describe the six-degrees-of-freedom (6-DOF) dynamics of a marine vessel, encompassing both translational and rotational behavior (*Y* denotes the translational forces, and *N* denotes the rotational moments with respect to an axis). In these equations and the subsequent equations in this section, M,C(v), and *D* are the inertia, Coriolis centripetal, and damping matrices. u,v, and *r* are the surge, sway, and yaw velocities, $\tau_{u}$ is the control force in surge, while δ is the rudder angle; therefore, $Y_{\delta }\delta$ and $N_{\delta }\delta$ are the rudder force and moment that affects the sway and yaw. The system can be represented as follows:

(2) $$\begin{array}{cc} \; & M\dot{v}+C\left(v\right)\;v+D\;v=\tau ,\\ \; & v=\left[\begin{array}{c} u\\ v\\ r \end{array}\right],\tau =\left[\begin{array}{c} t_{u}\\ Y_{\delta }\;\delta \\ N_{\delta }\;\delta \end{array}\right]. \end{array}$$

The matrices M,C(v), and *D* can be expressed as in the following, with parameters that differ from boat to boat:

(3) $$\begin{array}{cc} \; & M=\left[\begin{array}{ccc} m_{11} & 0 & 0\\ 0 & m_{22} & m_{23}\\ 0 & m_{32} & m_{33} \end{array}\right],D=\left[\begin{array}{ccc} d_{11} & 0 & 0\\ 0 & d_{22} & d_{23}\\ d_{32} & 0 & d_{33} \end{array}\right],\\ \; & C\left(v\right)=\left[\begin{array}{ccc} 0 & -\left(m_{22}\;v+m_{23}\;r\right) & -m_{11}\;u\\ m_{22}\;v+m_{23}\;r & 0 & 0\\ m_{11}\;u & 0 & 0 \end{array}\right]. \end{array}$$

While these equations provide a comprehensive description of vessel motion, their nonlinear and coupled nature often makes them computationally expensive for real-time path planning and control applications. Consequently, numerous studies have proposed simplified kinetic models to reduce computational complexity while maintaining sufficient accuracy. Among these, the Nomoto model has gained prominence due to its simplicity and effectiveness for underactuated vessels [3,17,18,19,20].

#### 2.2.1. The Nomoto Model

The Nomoto model (Equation (4)) is a single-input–single-output (SISO) transfer function that models the steering input to the yaw rate of a boat. Refs. [17,21,22] have all proposed using the International Marine Organization (IMO) standard maneuvering tests [23] to conduct system identification (SID) for the Nomoto model. Based on the findings from [17], this research would focus on the second-order Nomoto model, which is shown to be more in line with the actual heading of a ship than the first-order Nomoto model.

Being a second-order linear differential equation, the Nomoto model has some advantages, which include (1) being identifiable with data collected from GPS using linear system identification methods, (2) using only four parameters, and (3) using direct user input as the model input. The main drawback of the Nomoto model is that it does not account for drift. Even though the problem of modeling drift has been addressed in the form of modified Nomoto models such as the one proposed in [24,25], the authors determined that oyster harvesting trajectories should be planned based on Boustrophedon Cellular Decomposition (BCD).

Bottom-cultured oysters are typically grown on a layer immediately above the sea floor, deriving their nutrition primarily from suspended algae [26]. Consequently, effective oyster dredging requires the vessel to operate within a narrow speed window. Sailing too slowly drives the dredge excessively deep into the substrate, entraining rocks and debris, whereas sailing too quickly causes the dredge to skim the surface and miss oysters. Thus, precise, dynamic control of boat speed is essential to maintain a constant dredge depth and optimize harvest efficiency.

Furthermore, in the Chesapeake Bay and similar regions, bottom-cultured oysters are grown in nearshore waters that are shallower than 15 feet. Harvesting is typically carried out under calm conditions, when wind speeds remain below 10 knots, so that the vessels remain stable. Under these practical constraints, boats effectively operate at an almost constant velocity with negligible drift, justifying the Nomoto model’s assumption of a steady speed and no drift.

(4) $$\begin{array}{cc} \; & \frac{\omega (s)}{\delta (s)}=\frac{KT_{3}s+K}{T_{1}T_{2}s^{2}+\left(T_{1}+T_{2}\right)s+1} \end{array}$$

If these models are to be applied to path planning or motion planning methods, they would need to be combined with another model to represent the configuration space (or C space) of the coverage path planning problem ($R^{2}\times \left[-\pi ,\pi \right]$, also known in motion planning as the Special Euclidean group in two dimensions (SE(2))). Previous research has averted this problem by controlling the Nomoto model and using metrics based solely on the yaw (θ) rather than x,y,θ [27,28,29]. This method is an acceptable compromise for designing some less complex path planning systems. In the case of full-coverage path planning, instead of having only one pair of start and end positions, multiple reference points need to be consistently reached in succession. This necessitates a more direct modeling approach. The Krasowski model, which directly maps control inputs to spatial dynamics in SE(2), provides a suitable foundation.

#### 2.2.2. The Krasowski Model

A close fit of a pre-existing model for path- and motion planning is the hydrodynamic boat model developed by Krasowski et al. (Equation (5)), which predicts a vessel’s position and velocity in the SE(2) space [16] from applied forces along the x,y axes and a moment about the *z* axis ($F_{x},F_{y},M_{z}$).

The equation of the Krasowki Model is given as follows:

(5) $$\begin{array}{cc} \; & x_{1}=x,x_{2}=y,x_{3}=\theta ,x_{4}=v,x_{5}=v_{l},x_{6}=\omega \\ \; & u_{1}=F_{f},u_{2}=F_{l},u_{3}=M_{z}\\ \; & \left[\begin{array}{c} {\dot{x}}_{1}\\ {\dot{x}}_{2}\\ {\dot{x}}_{3}\\ {\dot{x}}_{4}\\ {\dot{x}}_{5}\\ {\dot{x}}_{6} \end{array}\right]=\left[\begin{array}{c} \left[\begin{array}{ccc} \cos x_{3} & -\sin x_{3} & 0\\ \sin x_{3} & \cos x_{3} & 0\\ 0 & 0 & 1 \end{array}\right]\left[\begin{array}{c} x_{4}\\ x_{5}\\ x_{6} \end{array}\right]\\ M^{-1}\left(-C(\nu )\left[\begin{array}{c} x_{4}\\ x_{5}\\ x_{6} \end{array}\right]-D\left[\begin{array}{c} x_{4}\\ x_{5}\\ x_{6} \end{array}\right]+\left[\begin{array}{c} u_{1}\\ u_{2}\\ u_{3} \end{array}\right]\right)) \end{array}\right] \end{array}$$

In Equation (5), $M\in R^{3\times 3}$ is the combined mass–inertia matrix of the vessel, and $C\left(\nu \right)\in R^{3\times 3}$ is the Coriolis–centripetal matrix, which is a function of the body-fixed velocity vector:

$$\nu ={\left[\begin{array}{ccc} x_{4} & x_{5} & x_{6} \end{array}\right]}^{T},$$

where $D\in R^{3\times 3}$ is the linear and quadratic damping matrix. These matrices capture, respectively, the vessel’s inertial properties, the coupling between translational and rotational motions, and hydrodynamic drag. Equation (5) then reads as follows:

$$\dot{x}\;=\;\left[\begin{array}{c} R(\theta )\;\nu \\ M^{-1}\left(-\;C(\nu )\;\nu \;-\;D\;\nu \;+\;u\right) \end{array}\right],$$

where

$$x={\left[\begin{array}{cccccc} x_{1} & x_{2} & x_{3} & x_{4} & x_{5} & x_{6} \end{array}\right]}^{T},\;u={\left[\begin{array}{ccc} u_{1} & u_{2} & u_{3} \end{array}\right]}^{T},$$

and

$$R\left(\theta \right)=\left[\begin{array}{ccc} \cos\theta & -\sin\theta & 0\\ \sin\theta & \cos\theta & 0\\ 0 & 0 & 1 \end{array}\right]$$

describe the rotation from a body-fixed to inertial frame. This notation makes clear that *C* depends on the current velocity vector ν, and that M, *C*, and D are all constant (or slowly varying) matrices, determined by the vessel’s geometry and hydrodynamic coefficients.

While this formulation works naturally for vessels that can command forces and moments directly—such as those with omnidirectional or water-jet thrusters—typical fishing boats are underactuated, relying on a single propeller and rudder. In their case, the primary control inputs are the propeller thrust and rudder angle rather than raw force vectors, so an additional mapping is required to convert those inputs into the equivalent $F_{x}$, $F_{y}$, and $M_{z}$. To capture this, one needs a hybrid framework that augments the Krasowski maneuvering model with a steering model (for the rudder) and, if desired, a Dubins-style kinematic layer for high-level planning.

#### 2.2.3. The Davidson and Schiff Model and Asfihani Approach

Ref. [30] provided a similar framework to what this research is proposing. They first calculated an optimal path based on the Dubins model, after which they designed model predictive controls based on the Davidson and Schiff model [20].

The Davidson and Schiff model (Equation (6)) is a model that maps the rudder angle to the sway(*v*), surge($u_{0}$), and yaw(*r*) velocities, and which is more extensive than the Nomoto model. However, this would result in more complicated system identification, since the sway and surge velocities would also have to be measured and used for system identification. This requires more tuning and equipment than identifying the Nomoto model, which this research has shown in Section 3.3 could be achieved with a simple localization system such as a single camera or GPS.

The main drawback of Asfihani’s approach is that since the planning is carried out offline first and then controlled, the approach is restricted to offline static path planning. Therefore, the approach proposed in this research aims to combine the Dubins model with the Nomoto model to allow for methods that require online dynamic planning and real-time feedback, such as the methods developed in [5,7].

(6) $$\begin{array}{ccc} \; & M\dot{\mu }+N\left(u_{0}\right)\mu =b\delta ,\; & \mu ={\left[\begin{array}{cc} v & r \end{array}\right]}^{T}\\ \; & M=\left[\begin{array}{cc} m-Y_{v} & mx_{G}-Y_{r}\\ mx_{G}-N_{v} & I_{z}-N_{r} \end{array}\right],\; & N\left(u_{0}\right)=\left[\begin{array}{cc} -Y_{v} & mu_{0}-Y_{r}\\ -N_{v} & mx_{G}u_{0}-N_{r} \end{array}\right] \end{array}$$

### 2.3. Motion Primitives for Boats

The calculation of motion primitives for marine vessels from heading and Cartesian coordinates has been extensively studied, with most research either focusing on ensuring compliance with the International Regulations for Preventing Collisions at Sea (COLREGs), as established by the International Maritime Organization (IMO) [31,32,33], or on autopilot systems. Consequently, existing studies primarily focus on developing motion primitives and control strategies that optimize path planning to avoid collisions while maintaining the intended trajectory of a vessel [34]. The fundamental challenge in these approaches lies in modeling decision-making and the subsequent controls between two or more vessels.

In contrast, the operational dynamics of fishing vessels, particularly those involved in oyster harvesting, differ significantly from those of conventional marine navigation. Typically, a single fishing boat operates within a designated lease area, thereby minimizing complex interactions between vessels. However, to enhance collection efficiency, future work will explore coordinated control of multiple boats operating within the same lease. This shift introduces new considerations in the development of motion primitives, particularly for cooperative path planning and task allocation.

Another critical factor in oyster harvesting, which has received little to no attention in the existing research, is the restriction of starboard(right)-turn-only movement. Since the dredge is deployed on one side of the boat, typically on the starboard side, the vessel must execute turns in that direction to prevent damage. Although primitive motion calculations that could accommodate turn constraints have been developed for aerial vehicles, particularly quadcopters [9,35,36], the application of such constraints to marine vessels remains largely unexplored. In particular, quadcopters exhibit complex, high-degree-of-freedom dynamics, whereas fishing boats operate under simpler constraints. Given this distinction, the authors propose modeling boat movement using the Nomoto model and computing starboard-turn-only motion primitives based on the Dubins model. This approach ensures a more practical and computationally efficient motion planning framework, tailored to the specific requirements of oyster harvesting operations. A summary comparison of the methods introduced in this section and their roles in motion planning is provided in Table 1.

## 3. Materials and Methods

### 3.1. General Definitions and Assumptions

The definitions of variables in this section are as follows: *v* denotes the forward velocity of the boat; ω denotes the angular velocity of the boat; x,y denotes the 2 axes of the coordinate system; X,Y, and *U* are the state, output, and input vectors of the proposed model; and the matrices A,B,C,D are the system matrices defining the state space representation:

$$\dot{X}=AX+BU,Y=CX+DU$$

Before deriving the hybrid model, we state our key assumptions and constraints. We assume that the vessel operates at a nearly constant forward speed,

$$v\;\in \;[\;v_{0}-\varepsilon ,\;v_{0}+\varepsilon \;],$$

where the nominal speed $v_{0}$ is set by mission requirements and ε is a small tolerance. The justification behind this irregular assumption of constant velocity was stated in Section 2.2.1. The steering command is normalized as

U∈[−1,1],

with full-scale deflection at the actuator limits. We neglect higher-order hydrodynamic effects, wave disturbances, and current drift, so that the combined Dubins–Nomoto dynamics remain smooth and twice differentiable over the trajectory of interest. For the linearized model, we require (i) that the system vector field is continuously differentiable in a neighborhood of each operating point $X_{p}$, and (ii) that both states and inputs remain bounded so that local Jacobian linearization accurately captures the true dynamics [37]. These assumptions define the domain of validity for both the nonlinear hybrid and its linear approximation.

### 3.2. Model Derivation

**Figure 3:**
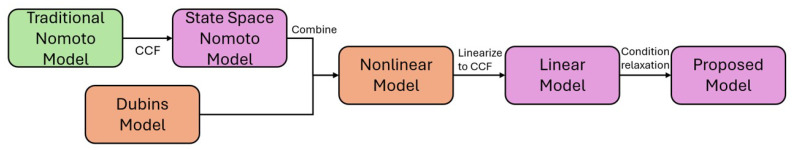
Overview of the model derivation process. Box colors indicate the form of the underlying equations: green represents transfer functions, orange denotes nonlinear models, and plum corresponds to linear models.

In the development of autonomous systems for aquaculture, accurate and flexible modeling of vessel dynamics is essential for effective navigation and control. Traditional models such as the Dubins and Nomoto models each offer useful properties—geometric simplicity and hydrodynamic realism, respectively—but fall short when applied independently in complex aquatic environments, as mentioned in Section 1. To address this limitation, we derive a hybrid model that integrates the kinematic structure of the Dubins model with the dynamic steering behavior captured by the Nomoto model (as shown in Figure 3). This combined formulation enables direct mapping from control inputs to vessel motion in SE(2) space while supporting multi-input–multi-output (MIMO) system extensions. The resulting model not only accommodates advanced control strategies but also lays the groundwork for incorporating additional components such as dredge dynamics, making it highly applicable to the evolving demands of aquaculture automation.

#### 3.2.1. Nonlinear Model

**Theorem** **1** (Controllable Canonical Form (CCF))**.** *Given the transfer function*

$$G\left(s\right)=\frac{b_{0}+b_{1}s+b_{2}s^{2}+\ldots +b_{n-1}s^{n-1}}{s^{n}+a_{1}s^{n-1}+a_{2}s^{n-2}+\ldots +a_{n}}$$

*the corresponding state space representation in CCF is as follows:*

$$\begin{array}{ccc} \; & \dot{x}=Ax+Bu,\\ \; & y=Cx+Du\\ \; & A=\left[\begin{array}{ccccc} 0 & 1 & 0 & \ldots & 0\\ 0 & 0 & 1 & \ldots & 0\\ \vdots & \vdots & \vdots & \ddots & \vdots \\ 0 & 0 & 0 & \ldots & 1\\ -a_{n} & -a_{n-1} & -a_{n-2} & \ldots & -a_{1} \end{array}\right],\; & B=\left[\begin{array}{c} 0\\ 0\\ \vdots \\ 0\\ 1 \end{array}\right]\\ \; & C=\left[\begin{array}{ccccc} b_{0} & b_{1} & b_{2} & \ldots & b_{n-1} \end{array}\right],\; & D=0 \end{array}$$

*The full proof is provided in Appendix B.*

Since the Dubins model is already expressed in a state space form, the Nomoto model is converted from its transfer function representation (Equation (4)) into a state space form using Theorem 1, with *U* as the normalized steering input. In contrast, *z* denotes the dummy state vector for the CCF form of the Nomoto model, while $K,T_{1},T_{2},T_{3}$ are the parameters for the Nomoto model.

(7) $$\begin{array}{cc} \; & \dot{\eta }=f\left(\eta ,u\right)=\left[\begin{array}{c} v\cos(\eta_{3})\\ v\sin(\eta_{3})\\ \omega \end{array}\right] \end{array}$$

(8) $$\begin{array}{cc} \; & z=\left[\begin{array}{c} z_{1}\\ z_{2} \end{array}\right],z_{2}={\dot{z}}_{1}\\ \; & \dot{z}=\left[\begin{array}{cc} 0 & 1\\ -\frac{1}{T_{1}T_{2}} & -\frac{T_{1}+T_{2}}{T_{1}T_{2}} \end{array}\right]z+\left[\begin{array}{c} 0\\ 1 \end{array}\right]U\\ \; & \omega =\left[\begin{array}{cc} \frac{K}{T_{1}T_{2}} & \frac{KT_{3}}{T_{1}T_{2}} \end{array}\right]z \end{array}$$

Equation (7) represents the Dubins model, and Equation (8) is the Nomoto model in a state space form. Then the states *z* and η are concatenated o form the state space model with a new state *X*:

(9) $$\begin{array}{cc} \; & \dot{X}=\gamma \left(X,U\right)=\left[\begin{array}{c} X_{2}\\ -\frac{1}{T_{1}T_{2}}X_{1}-\frac{T_{1}+T_{2}}{T_{1}T_{2}}X_{2}+U\\ v\cos(X_{5})\\ v\sin(X_{5})\\ \frac{K}{T_{1}T_{2}}X_{1}+\frac{KT_{3}}{T_{1}T_{2}}X_{2} \end{array}\right]\\ \; & U=\frac{\delta }{\max(\delta )},x=X_{3},y=X_{4},\theta =X_{5} \end{array}$$

The resulting combined model also inherits a lot of advantages from both the Nomoto model and the Dubins model, with the main disadvantage being that the model is nonlinear. However, this is a necessary compromise to account for multiple state variables and the nonlinear Dubins model.

The model’s support for MIMO systems is an essential characteristic. As research on dredge localization is still ongoing, the dredge dynamics model will need to be appended to the model in the future to pinpoint the dredge underwater accurately; therefore this formulation also serves the purpose of enabling MIMO, which allows other dynamic models to be concaternated as well. Currently, of the models mentioned in Section 2, only the Krasowski model supports MIMO systems. Additionally, MIMO systems could later be expanded to include other factors, such as environmental variables, within the model. Therefore, sacrificing linearity in exchange for MIMO support is considered a necessary trade-off.

In summary, this boat model would have the capability to fulfill the main objective of directly mapping the steering input to the SE(2) space. The model is also formalized so that previous methods designed for both the Nomoto and Dubins models can still be used, a feature that this research draws heavily upon during tests and analysis. For example, the RSR maneuver in the Dubins model, which can be proven to be an R-geodesic based on the results in [8], is the optimal maneuver, given that the boat only has a starboard-turn-only condition.

#### 3.2.2. Linearized Model

The main purpose of a new boat model is to enhance boat control; therefore this research provides some analysis within control theory to explain the properties and constraints of the model. Therefore, a common method used to analyze nonlinear systems is to perform local linearization by finding the Jacobian of the system. However, even though the combined model does not have any equilibrium points, the local linearization of the system only exists due to the fact that the following of a single continuous trajectory from start to end satisfies the criteria mentioned in Section 3.1. Therefore, local linearization can be conducted around all points on the collection trajectory instead of just the equilibrium points. The point on the collection trajectory around which the system is linearized is defined as $X_{p}={\left[\begin{array}{ccccc} X_{p1} & X_{p2} & X_{p3} & X_{p4} & X_{p5} \end{array}\right]}^{T}$. The resulting locally linearized system of Equation (9) would be as follows:

(10) $$\begin{array}{ccc} \; & \dot{X}=AX+BU,\\ \; & Y=CX+DU,\\ \; & A=\left[\begin{array}{ccccc} 0 & 1 & 0 & 0 & 0\\ -\frac{1}{T_{1}T_{2}} & -\frac{T_{1}+T_{2}}{T_{1}T_{2}} & 0 & 0 & 0\\ 0 & 0 & 0 & 0 & -v\sin(X_{p5})\\ 0 & 0 & 0 & 0 & v\cos(X_{p5})\\ \frac{K}{T_{1}T_{2}} & \frac{KT_{3}}{T_{1}T_{2}} & 0 & 0 & 0 \end{array}\right], & B=\left[\begin{array}{c} 0\\ 1\\ 0\\ 0\\ 0 \end{array}\right],\\ \; & C=\left[\begin{array}{ccccc} 0 & 0 & 1 & 0 & 0\\ 0 & 0 & 0 & 1 & 0\\ 0 & 0 & 0 & 0 & 1 \end{array}\right], & D=\left[\begin{array}{c} 0\\ 0\\ 0 \end{array}\right] \end{array}$$

One criterion that is necessary for control and motion planning methods such as [5,7] to work is that the system needs to be completely controllable.

Using the MATLAB (version R2022b) ctrb(A, B) function, it can be calculated that the rank of the controllability matrix is 4 and therefore not completely controllable. This is to be expected, since the velocity is given as a constant due to the constraints of the Nomoto model. Therefore, to increase the controllability this research suggests that instead of assuming that the velocity is constant as in the Nomoto model, the conditions are relaxed so that perturbations in velocity are allowed. The output matrix C simply extracts the vehicle’s SE(2) pose, so those three states are the only ones observed. The remaining states (e.g., velocities or biases) are left unobservable by design, since this model’s sole purpose is to drive rudder commands directly from the pose. In other words, the system is intentionally not fully observable to focus on mapping SE(2) coordinates to control inputs.

(11) $$\begin{array}{cccc} \; & U\in [-1,1], & v\in [v_{0}-\epsilon ,v_{0}+\epsilon ], & u={\left[\begin{array}{cc} U & v \end{array}\right]}^{T} \end{array}$$

The resulting linearized model is then developed to be the following:

(12) $$\begin{array}{ccc} \; & A=\left[\begin{array}{ccccc} 0 & 1 & 0 & 0 & 0\\ -\frac{1}{T_{1}T_{2}} & -\frac{T_{1}+T_{2}}{T_{1}T_{2}} & 0 & 0 & 0\\ 0 & 0 & 0 & 0 & -v\sin(X_{p5})\\ 0 & 0 & 0 & 0 & v\cos(X_{p5})\\ \frac{K}{T_{1}T_{2}} & \frac{KT_{3}}{T_{1}T_{2}} & 0 & 0 & 0 \end{array}\right],\; & B=\left[\begin{array}{cc} 0 & 0\\ 1 & 0\\ 0 & \cos(X_{p5})\\ 0 & \sin(X_{p5})\\ 0 & 0 \end{array}\right],\\ \; & C=\left[\begin{array}{ccccc} 0 & 0 & 1 & 0 & 0\\ 0 & 0 & 0 & 1 & 0\\ 0 & 0 & 0 & 0 & 1 \end{array}\right],\; & D=\left[\begin{array}{cc} 0 & 0\\ 0 & 0\\ 0 & 0 \end{array}\right] \end{array}$$

By deriving a nonlinear MIMO system that is capable of steering directly in the SE(2) space, the model is designed to accommodate complex maneuvering tasks such as dredge positioning and full-coverage navigation. Its structure supports modular expansion to include additional dynamics, such as underwater dredge behavior or environmental disturbances, making it highly adaptable to diverse aquaculture scenarios. Crucially, the research introduces a linearized version of the model—via local Jacobian analysis along trajectory points—that is compatible with modern control techniques, despite lacking equilibrium points (a preliminary sample is shown in Appendix A). Relaxing the constant velocity constraint improves controllability, enabling integration with advanced motion planning algorithms. This comprehensive approach provides a foundation for more intelligent, efficient, and scalable automation in marine farming operations, ultimately contributing to the sustainability and productivity of aquaculture systems.

### 3.3. Experimentation

#### 3.3.1. Hardware and Setup

**Figure 4:**
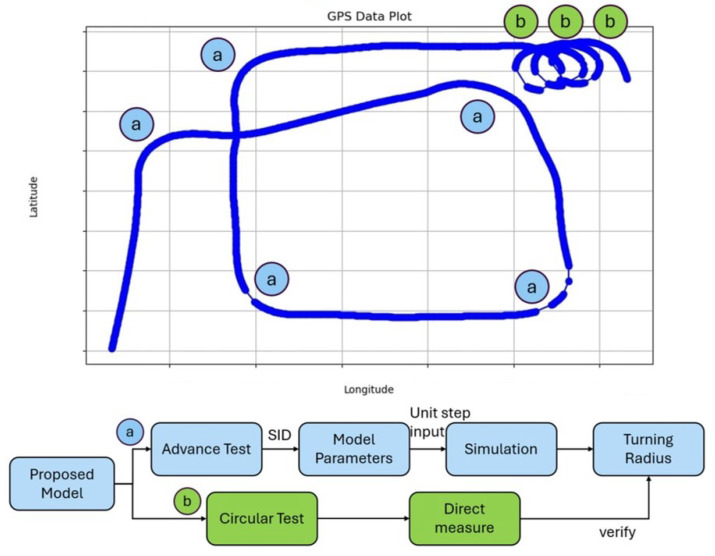
Starting in the south-west and ending in the north-east, the boat conducted five advance tests and three circle tests. The tests involved in this figure are defined as a set of experiments. These experiments were then used to conduct SID for the proposed model. The wind speed during the time of this experiment was 7 knots.

Modifying the system identification utilized by [17], this research proposes using the advance maneuver defined by the IMO. The official definition is as follows: advance is the distance traveled in the direction of the original course by the midship point of a ship from the position at which the rudder order is given to the position at which the heading has changed 90° from the original course [23]. Due to the limited view angle of the data collection setup and for consistency reasons, the circle tests utilized by [17] were conducted as verification tests using an external localization method (as shown in the flow chart in Figure 4). The current test with our research boat utilizes an accurate GPS system for localization in the form of a Trimble (Westminster, CO, USA) BX992 RTX GNSS system.

**Figure 5:**
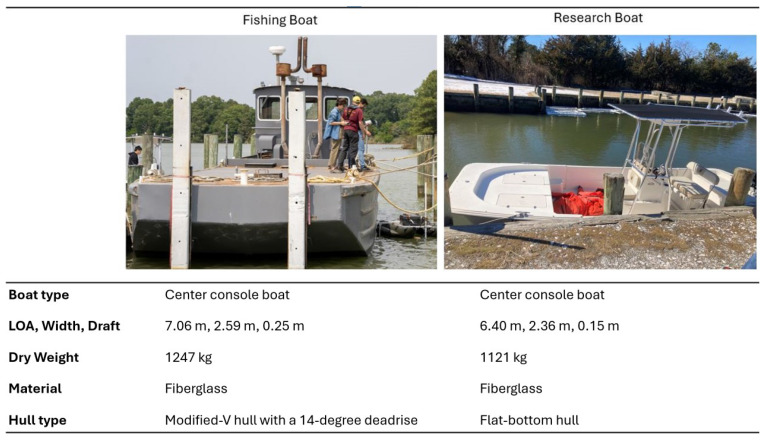
General boat specs of the research boat used for testing compared to an actual oyster harvesting boat.

The experiment was conducted at the Horn Point Laboratory (HPL), University of Maryland Center for Environmental Science (UMCES), in Cambridge, MD, USA, using a research boat, whose specifications are listed in Figure 5. The test site had an average depth of 15 feet, which, based on [38], classifies it as deep water. Consequently, the effects of depth are considered negligible. The primary sources of disturbance in this experiment were wind and waves. To account for wind conditions, the authors obtained and recorded wind speed data from the National Data Buoy Center, specifically from station CAMM2, which is located directly at the test site. On the day of testing, wind speeds were recorded at 1.8 knots NNW. The trials were designed and conducted specifically with oyster harvesting tasks and the environment of the test site in mind. For example, oyster farmers in the Chesapeake Bay typically avoid harvesting operations when wind speeds exceed 10 knots. Details regarding the specifications of an actual fishing boat and the research boat used in this experiment are also shown in Figure 5 as a reference.

#### 3.3.2. Experimental Process

First, an advance test is conducted, and the trajectory is documented. The boat’s yaw is then computed by analyzing the difference between each point along the path and its relative orientation compared to the initial heading. Using the recorded start times of each turn, a set of input and output signals for the Nomoto model are derived. The parameters of the Nomoto model are then identified using MATLAB’s System Identification Toolbox.

Once the parameters are determined, they, along with the velocity, are incorporated into the boat model, which is then simulated using a unit step input. The resulting output is used to determine the minimum turning radius.

Next, the minimum turning radius and the designated start and end points are input into a starboard-turn-only path planning algorithm. This algorithm computes the nominal trajectory and generates a sequence of driving instructions, guiding the operator through straight movements and starboard turns over specified time intervals. The complexity of the path planning algorithm warrants its own dedicated research study.

## 4. Results

### 4.1. System Identification

Due to the properties of the Dubins model, as mentioned in Section 2.1, the minimum turning radius can be used to verify the model’s validity sufficiently. The turning radius is then compared to the ground truth by driving the boat in three consecutive circles and finding the real-world radius of the smallest circle. The ground truth shows that the minimum turning diameter for a circle is 12.44 m (radius 6.22 m).

The authors structured a standardized set of IMO maneuvers into a single experimental sequence (Figure 4). First, the driver is instructed to move forward until any noticeable sideways momentum dissipates. Then, an advance maneuver is performed, which is repeated five times. Following this, the driver completes three consecutive full-circle maneuvers. This approach allows for the completion of five advance tests and three circular tests within approximately 30 min, optimizing both fuel efficiency and time management.

In total, six sets of tests for system identification are conducted by a human control without real-time yaw readings. Thus, it can be seen in all tests that the actual conducted turn is not exactly 90 degrees. Given this inaccuracy during the tests, as well as wind and wave disturbances, the results show this. Therefore, they show the robustness of the system identification.

After system identification was conducted, the data was first processed, since data loss from the GPS rendered parts of the data unusable. The authors used GNSS data that were provided directly through the RTX GNSS BX992 manufactured by Trimble (Westminster, CO, USA), and no additional filtering or interpolation was used in system identification. Out of the 30 repetitions (six sets) of the IMO advance test, 18 were usable and identified using the MATLAB System Identification Toolbox. The parameters were then implemented on the boat model and tested on a unit-step input. The results were then compiled into the turning radius. Based on the system identification results, the boat’s average turning radius is 6.13 m, with a standard deviation of 0.327 m. Compared with the ground truth, the error is 0.09 m, which is a 1.45% error.

Above all, as all equipment used in the experiments is stated in Section 3.3, all experimentation can be conducted using equipment that is standard in the fishing industry setup without any additional purchases by the fisherman. Therefore, the cost efficiency of this approach significantly increases the likelihood that the industry will adopt the system.

### 4.2. Trajectory Following

In addition to the Dubins turning radius, another critical factor is the placement of the dredge on the side of the fishing boat, which restricts turning in that direction. Turning the opposite way risks the towing cables of the dredge being scrapped against the hull, potentially damaging its protective coating. Therefore, an essential consideration in oyster aquaculture path planning is ensuring that the boat can only turn in one direction. Discussions with oyster farmers indicate that the dredge is typically positioned on the starboard side of the boat.

**Figure 6:**
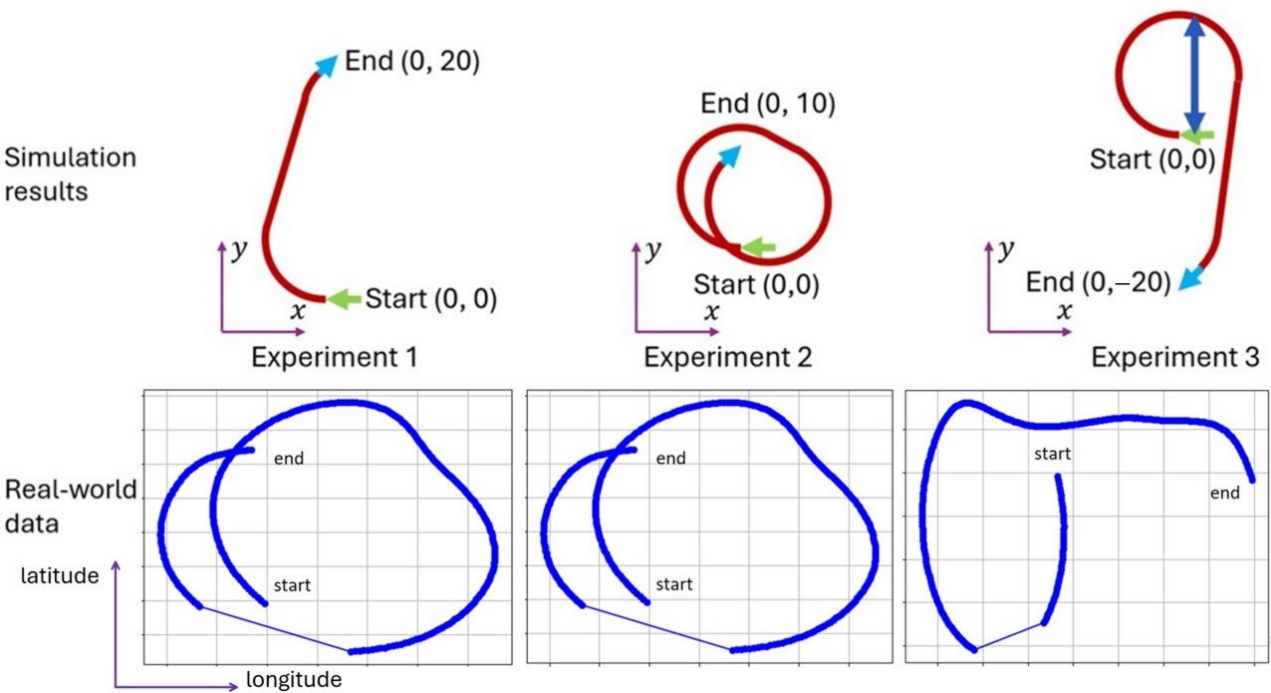
The (**top row**) illustrates the simulation results (with the turning diameter being roughly 12.5 m and the length overall (LOA) of the boat being roughly 6.4 m) for each planned trajectory, while the (**bottom row**) presents the corresponding real-world GPS traces collected at Horn Point Laboratory. Wind conditions during testing were stable (1 knot). The full MSE results and trajectory error analysis are available in Section 4.3.

In terms of Dubins path planning, this means that of the six Dubins maneuvers defined, only the Right–Straight–Right(RSR) maneuver is available. In terms of the possible types of maneuvers in SE(2), they can be roughly categorized into three categories: the end point is on the right side of the start point with a parallel distance that is larger than the minimum turning radius (Experiment 1), the end point is on the right side of the start point but with a parallel distance that is smaller than the minimum turning radius (Experiment 2), and the end point is on the port side of the start point (Experiment 3) (Figure 6).

Once the system identification (SID) of the boat is completed and the turning radius is determined, driving instructions for the required maneuvers are generated. If the boat is traveling at a speed of 5 mph, the driving instructions for each experiment are calculated as maneuver timings:

1. Starboard 5.5 s, straight 4.5 s, starboard 1 s;
2. Starboard 13 s, straight 2 s, starboard 21 s;
3. Starboard 19.5 s, straight 10 s, starboard 2 s.

The driver was provided only with the starting coordinates, the corresponding set of instructions, and the yaw angle at the endpoint for easier driver reference. To clarify, no timing aid (e.g., stopwatch or audible cues) was used; the driver relied on internal judgment. A maximum of two practice runs were allowed per trajectory, and the same individual performed all trials to eliminate inter-subject variability. This consistency controls for driver variation but likely contributes to most of the errors throughout all experiments. All maneuvers were executed manually without autopilot; the model was used solely to generate time-based driving instructions. This setup reflects current aquaculture practices and helps isolate where human error may be reduced through automation in future work.

For each experiment, the actual trajectory of the boat is then recorded over 10 trials and compared to the nominal trajectory to evaluate performance and accuracy. The primary metric used for this comparison is the distance offset from the intended endpoint.

It is important to note that the experiments were conducted in order of increasing simplicity, with Experiment 2 being executed first and Experiment 1 last. This sequencing was intentionally designed to mitigate the potential influence of driver fatigue on the performance of more complex maneuvers. By addressing the most demanding trajectories early in the testing sequence, the study aimed to preserve the integrity and comparability of the trajectory following results. Based on initial expectations, error was anticipated to correlate positively with trajectory complexity—with Experiment 2 being the most error-prone and Experiment 1 exhibiting the least deviation.

**Figure 7:**
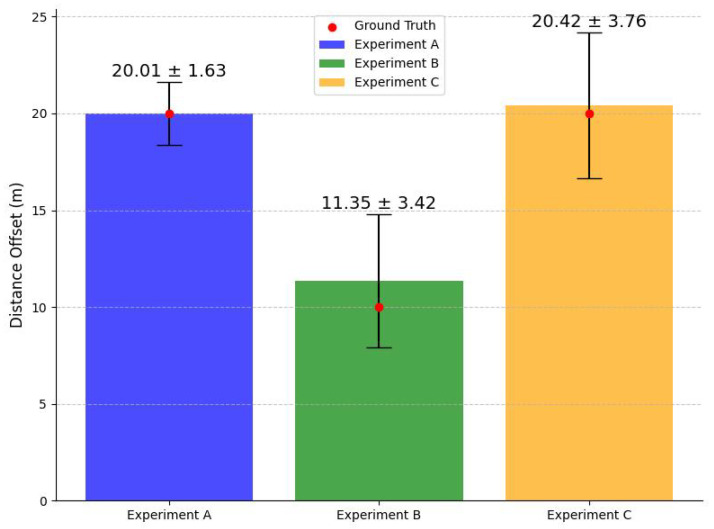
Colored barchart and error bars representing the sample mean ± standard deviation in meters for each experiment; the red dots indicate the ground truth distances. Full numerical results and error breakdowns are provided in Section 4.3. The ground truth values correspond to target displacements defined in Figure 6.

Based on the experiments, the average offset and standard deviation of Exp.1, Exp.2, and Exp.3 are 0.01, 1.35, and 0.42 m, respectively, with standard deviations of 1.63, 3.42, and 3.76 m. These can be compiled as a 0.05%, 13.5%, 2.10% error, respectively. These results are plotted in Figure 7.

### 4.3. Analysis

The experimental results generally support the expectation that the level of errors increases with trajectory complexity. For the Euclidean distance, shown in Table 2, Experiment 1, the simplest maneuver, showed excellent performance, with an average offset of just 0.01 m, a very low 0.05% error, and a standard deviation of 1.63 m, resulting in a 95% confidence interval of [−1.156,1.176], indicating both high accuracy and consistency. Experiment 3, which was moderately complex, maintained a low average offset of 0.42 m and a 2.10% error, resulting in a 95% confidence interval of [−1.097,3.797], although it exhibited the highest variability, with a standard deviation of 3.76 m. However, considering the ground truth for Experiments 1 and 3 is 20 m and that for Experiment 2 is 10 m, it can be seen that Experiment 2 has a relatively higher standard deviation by proportion. Experiment 2, the most complex task, had the most significant average offset of 1.35 m and a high standard deviation of 3.42 m, resulting in a 95% confidence interval of [−2.270,3.110]. In absolute terms, its error is substantial but more comparable to Experiment 3 when normalized across different trajectory lengths. Note that the distances shown below are the absolute values, since it is a measure of distance.

The actual difference in Euclidean distances for each test is shown below; for clarity, results from Experiment 1 are classed as Group A, Experiment 2 as Group B, and Experiment 3 as Group C.

Mean Square Errors (MSEs) are a standard metric that is used to evaluate trajectory following performance (Shown in Table 3). However, the full coverage of oyster harvesting is to be planned out using Boustrophedon Cellular Decomposition (BCD, also known as the lawn mower pattern). Given that all collection trajectories are straight sections, the methods developed in this research are used for the transition segments in between line sections, and the MSE of the transition segments is less critical. Due to the limitations of our GNSS system, some data have significant gaps, with the recorded trajectories having up to 3–10 s gaps in the middle where no trajectories are recorded (see Figure 4, real-world data, and Experiments 2 and 3). Therefore, the MSE for the data where the data loss is less significant is shown; the experiments where data loss gaps are greater than 5% of the experiment duration are shown as ERR, whereas for experiments where data loss gaps are less than 5%, the lost data points are taken out of the nominal trajectory.

In terms of the entire trajectory, the Frechet distance is used. The Frechet distance is defined as the “minimal leash length while walking a dog”, which is the worst case deviation between two points on separate trajectories at the same moment of time without backtracking (Shown in Table 4). However, since the relative time frame of the trajectory also matters when calculating the Frechet distance compared to the Hausdorff distance, the data gaps would affect the Frechet distance significantly, and therefore experiments with data loss are also shown as ERR.

The difference in the set of points within the trajectory is compared in terms of the Hausdorff distance. The Hausdorff distance is defined as the worst case deviation between two sets of points in a metric space with a distance function that is the Euclidean distance (shown in Table 5).

Except for Experiment 8, the tests from Experiment 1 are mostly intact. However, all the experiments in Group B had significant data loss. The experiments from Group C land somewhere in between. Based on the MSE from Groups A and C, the average MSE from Group A was 1.849 m with a standard deviation of 1.420 m, and the average MSE from Group C was 4.100 m with a standard deviation of 0.911 m. The average Frechet distance from Group A was 5.085 m with a standard deviation of 0.035 m, and the average from Group C was 5.603 m with a standard deviation of 2.268 m. The results from the MSE are primarily in line with the expectations stated in Section 3.2. However, the results from the Frechet distance showcased a flaw in the calculations of the ground truth, which is that the acceleration included during the start of the turn is not modeled in the ground truth. The original assumptions are that turning trajectories will travel along the trajectory at a speed of $\frac{v}{\sqrt{2}}$. However, during testing, it was found that at the start of the turn, the speed drops significantly below the estimated speed, but then the driver recovers by gradually increasing the speed during the turn. This then causes a lag between the two trajectories, as shown by the consistency in the Frechet distances. This, however, causes significant problems when calculating the MSE and Hausdorff distance, since the majority of errors recorded using MSE and Hausdorff distance are close to the data loss points. This is evident when processing data from Group C, specifically experiments C2, C3, and C5, which have minor data losses of less than 5% of the entire trajectory. However, the Frechet distance is equal to the Hausdorff distance in these instances. This occurs because the maximum deviation happens when data loss occurs during the lag period, and therefore, the ground truth is already ahead of the traversed trajectory when the data loss occurs.

Through these experiments, the authors concluded that as the number of turns in a trajectory increases, the system becomes less tolerant to human timing errors and lag. GPS data show that the turns in Experiment 1 are generally accurate, whereas in the first turn of Experiment 3 and both turns in Experiment 2, the driver tends to overturn. These patterns suggest that increased maneuver complexity leads not only to more significant average errors but also to higher variability in performance. The results indicate that driver-induced errors are most pronounced during sharp or complex turning events. Consequently, the authors believe that a well-designed control system with autopilot functions could effectively compensate for these errors, which remains an important area for future work. Within the context of the experiment, Experiments 1 and 3 are considered successful, while Experiment 2, though less accurate, still produced results that a controller could reasonably correct. However, from the perspective of practical oyster harvesting, the maneuver used in Experiment 2 is deemed unrealistic, as the sharp turns would generate excessive momentum, posing safety risks due to unsecured equipment onboard.

## 5. Discussion

In summary, this study introduces a novel kinodynamic model that is tailored to precision oyster aquaculture, achieved through the integration of the Nomoto model, which is commonly used for ship maneuvering, with the Dubins path framework, which characterizes constrained turning trajectories. This hybrid formulation enables the direct mapping of steering inputs to the boat’s position and orientation in the planar SE(2) space, thereby eliminating the need for intermediate representations, such as the yaw rate or force components, that are typically used in traditional maritime models. This not only enhances controllability but also streamlines motion planning and trajectory tracking, enabling more efficient and accurate maneuver execution. The model was validated through a series of field experiments on a research vessel in the Chesapeake Bay, which demonstrated strong agreement between the planned and actual trajectories. Experiments involving varying maneuver complexities yielded low average offset errors and standard deviations—particularly in Experiments 1 and 3—indicating high consistency and robustness. Although Experiment 2, which involved the sharpest turns and shortest trajectory, exhibited the most significant error and variability, the deviation remained within tolerances that a closed-loop control system could feasibly address. Notably, the study highlights a practical constraint in oyster harvesting operations: sharp, aggressive turns, as required in Experiment 2, are not only mechanically demanding but also operationally hazardous due to the potential destabilization of unsecured gear. This observation underscores the importance of aligning maneuver strategies with real-world constraints.

Moving forward, future work will focus on embedding the proposed model into autonomous control frameworks and developing real-time, adaptive path planning algorithms that account for aquaculture-specific constraints—such as starboard-turn-only maneuverability, safe turning radii, and spatial coverage optimization—to improve operational safety, system autonomy, and overall harvesting efficiency in commercial oyster farming.

## Figures and Tables

**Table 1:** Comparison of proposed model with similar research.

Model Comparison	Input → Output	Relative Simplicity	Main Usage
Nomoto	Steering → Yaw Velocity	High	Single-Rudder–Propeller Boats
Dubins	Yaw Velocity → x,y Coordinates	High	Vehicles with Turning Radius
Davidson Schiff	Steering → Sway, Surge, Yaw Velocity	Low	Single-Rudder–Propeller Boats
Krasowski	x,y,z Forces → Velocity and Acceleration	Moderate	Jet Propulsion Boats
Proposed Method	Steering → x,y Coordinates	Moderate	Single-Rudder–Propeller Boats

**Table 2:** Euclidean distance in meters from ground truth for experiment groups A, B, and C.

Group	1	2	3	4	5	6	7	8	9	10
A	3.882	1.618	1.204	1.253	1.664	1.333	0.828	1.383	2.559	1.168
B	0.1186	4.2825	1.6596	3.8799	6.2845	1.2872	3.4276	4.5934	2.4979	2.6499
C	7.8421	0.0148	6.3975	0.9592	3.8577	2.2120	2.2539	0.7595	0.0169	0.0317

**Table 3:** MSE in meters from ground truth for experiment groups A, B, and C.

Group	1	2	3	4	5	6	7	8	9	10
A	4.319	1.296	1.569	1.999	2.339	1.029	0.092	ERR	3.944	0.048
B	ERR	ERR	ERR	ERR	ERR	ERR	ERR	ERR	ERR	ERR
C	ERR	4.721	4.767	ERR	2.812	ERR	ERR	ERR	ERR	ERR

**Table 4:** Frechet distance in meters from ground truth for experiment groups A, B, and C.

Group	1	2	3	4	5	6	7	8	9	10
A	5.054	5.055	5.062	5.137	5.069	5.137	5.107	ERR	5.091	5.049
B	ERR	ERR	ERR	ERR	ERR	ERR	ERR	ERR	ERR	ERR
C	ERR	4.651	8.191	ERR	3.968	ERR	ERR	ERR	ERR	ERR

**Table 5:** Hausdorff distance in meters from ground truth for experiment groups A, B, and C.

Group	1	2	3	4	5	6	7	8	9	10
A	3.085	1.618	1.204	1.253	1.664	1.333	0.828	ERR	1.201	1.168
B	ERR	ERR	ERR	ERR	ERR	ERR	ERR	ERR	ERR	ERR
C	ERR	4.651	8.191	ERR	3.968	ERR	ERR	ERR	ERR	ERR

**Figure A1:**
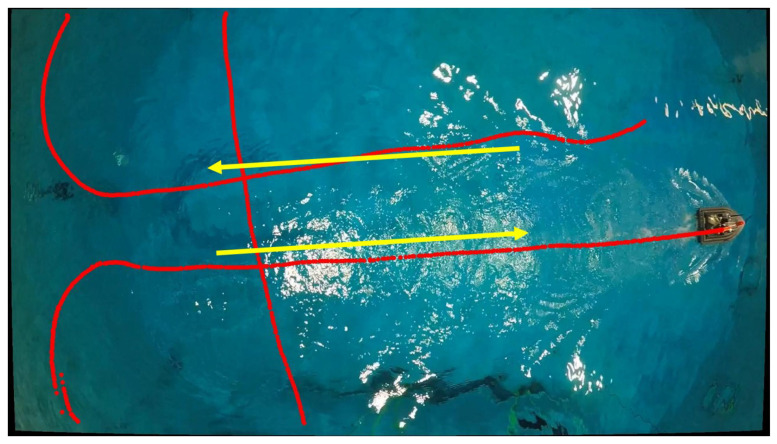
Sample experiment of RC boat trajectory following test. The goal is to travel along the yellow arrows (top (right to left) to (bottom (left to right)), which are overlayed on the driver’s monitor, given the driving instructions. The path that the boat has traversed has been superimposed onto the final image as the red line.

**Figure A2:**
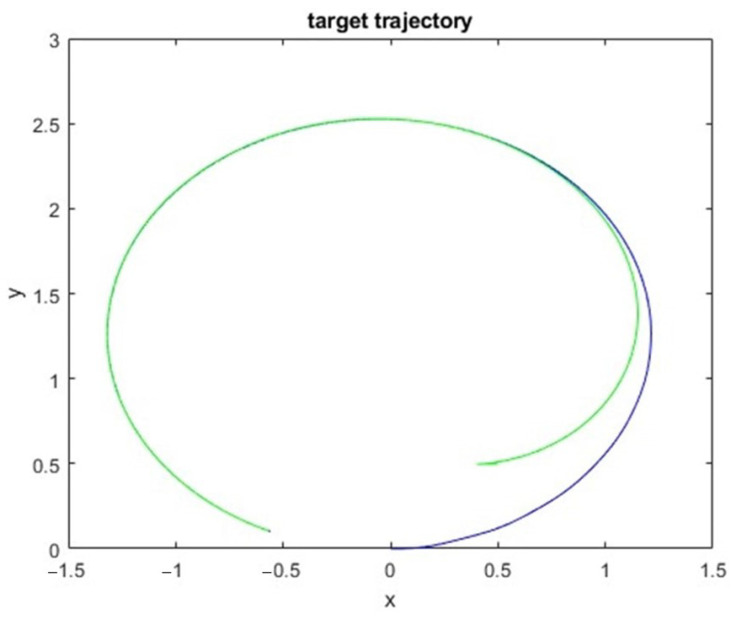
Sample simulation result of an LQR controller, implemented on the proposed model using the system identification results from the RC boat test. The blue trajectory is the nominal trajectory, starting at (0, 0), while the green trajectory is the controlled trajectory, with a starting point of (0.5, 0.5) and trying to correct back to the nominal trajectory.

## Data Availability

The datasets and code used within the context of this research will be made available on request.

## Abbreviations

The following abbreviations are used in this manuscript:

BCD	Boustrophedon Cellular Decomposition
CCF	Controllable Canonical Form
COLREG	International Regulations for Preventing Collisions at Sea
DOF	Degree-of-Freedom
FAO	United Nations Food and Agriculture Organization
GPS	Global Positioning System
GNSS	Global Navigation Satellite System
IMO	International Marine Organization
LOA	Length Overall
MSE	Mean Square Error
MIMO	Multi-input–Multi-output
RTK	Real-Time Kinematics
SE	Special Euclidean Group
SID	System Identification

## Appendix A. Preliminary Experiments

### Appendix A.1. Preliminary Experiment on an RC Boat

#### Appendix A.1.1. Hardware, Setup, and Assumptions

This preliminary test was conducted before the experiments in Section 2.3 and shared the same assumptions. The main differences are listed in the following paragraphs.

The data collection hardware in this experiment includes a GoPro Hero 5 manufactured by GoPro (San Mateo, CA, USA) mounted on an 8-foot (2.44 m)-high PVC pipe, looking down for localization and tracking. The boat used is a D16C Smart Remote Control Fishing Bait Boat. The specifications are 50 cm LOA, 26.5 cm width, and powered by twin propellers. Therefore, this experiment is considered to be scaled down approximately 30:1 and tested under idealized conditions.

The experiment was conducted within the Neutral Buoyancy Research Facility (NBRF) at the University of Maryland at College Park. The tank is 50 feet across and 25 feet deep. Compared to regular oyster harvesting, which involves a boat with a LOA of 23 feet (7.01 m), harvesting at a depth of around 15 feet (4.57 m), this test assumes that the depth and bathymetry are ignored.

#### Appendix A.1.2. Experimental Process and Results

To conduct system identification for the Nomoto model, the first test needed is a velocity test. By attaching a camera to the boat and calculating the number of frames between the boat starting and the impact on the other shore, given the distance between the starting point and the opposite shore, the velocity can be calculated. The boat is put on full throttle while held stationary before starting. The result is that, given the camera running at 60 frames per second (fps), the boat takes 1234 frames to travel from one end to the other. Given that the diameter of the tanks is 50 feet, the travel time of 20.57 s yields a velocity of 0.7380 m/s.

Then the advance test is conducted and recorded with a top-down camera. The boat yaw is then calculated by using the difference between each point on the path and the relative orientation compared to the initial orientation. Combined with the timing of the start time of each turn, a set of input and output signals of the Nomoto model can be calculated. Calculating the results using the Matlab System Identification Toolbox, the parameters for the Nomoto model can be found. After this, the parameters, along with the velocity, can be substituted into the Nomoto–Dubins model before running a unit step input. The resulting output is then used to determine the minimum turning radius.

By comparing 4 experiments (sample shown in Figure A1), the average turning radius of the boat is calculated to be 1.260 m, with a standard deviation of 0.02532 m. Compared with the verification data, the error is 0.056 m, which is a 4.255% error. However, the authors must state that the verification data used in this research is also not perfect, since there are intrinsic errors in verification data based on camera calibration and pixel resolution.

Due to hardware limitations, as shown in Figure A1, conducting path following with the RC boat is not feasible, since the boat is too small to fit a GPS that could track centimeter-level movement. Also, since flying a drone is not permitted in our facility, the field of view for a top-down camera is not sufficient to capture the entire trajectory.

### Appendix A.2. Preliminary Simulation of a Linear Quadratic Regulator (LQR)

An LQR controller is designed based on the following derivation of the Algebraic Riccati Equation (ARE) and the feedback input. The reference data used in the controller are denoted by _*ref*_. A,B are the state space matrices and *X* is the state vector defined in Equations (10) and (12). Q,R are the given control parameter matrices used to calculate *P* using the Algebraic Riccati Equation (ARE) before using *P* to calculate the control gain *K*. Detailed analysis and real-world controller testing are currently being conducted and are beyond the scope of this paper. Therefore, a sample simulation is shown in Figure A2. It is also noted that since the first two states are not observable, *Q* and *R* has been modified to minimize the simulated control signal. Then the first two columns of the feedback gain matrix *K* are set to 0 s.

(A1) $$\begin{array}{cc} \; & A^{T}P+PA-PBR^{-1}B^{T}P=-Q,\\ \; & u=u_{ref}-K\left(X-X_{ref}\right)\\ \; & K=R^{-1}B^{T}P \end{array}$$

## Appendix B. Generalized Controllable Canonical Form (CCF)

The following transfer function is given:

$$G\left(s\right)=\frac{b_{0}+b_{1}s+b_{2}s^{2}+\ldots +b_{n-1}s^{n-1}}{s^{n}+a_{1}s^{n-1}+a_{2}s^{n-2}+\ldots +a_{n}}$$

**Theorem** **A1** (Cayley–Hamilton Theorem)**.** *Let $A\in R^{n\times n}$ be a square matrix, and let ϕ(s)=det(sI−A) be its characteristic polynomial. Then the matrix A satisfies its own characteristic equation; that is,*

$$\phi \left(A\right)=A^{n}+a_{1}A^{n-1}+\ldots +a_{n-1}A+a_{n}I=0$$

*This implies that any power of A higher than n−1 can be written as a linear combination of lower powers:*

$$A^{k}=\sum_{i=0}^{n-1}\gamma_{i}^{\left(k\right)}A^{i},\forall k\geq n$$

The corresponding state space representation in Controllable Canonical Form is as follows:

$$A=\left[\begin{array}{ccccc} 0 & 1 & 0 & \ldots & 0\\ 0 & 0 & 1 & \ldots & 0\\ \vdots & \vdots & \vdots & \ddots & \vdots \\ 0 & 0 & 0 & \ldots & 1\\ -a_{n} & -a_{n-1} & -a_{n-2} & \ldots & -a_{1} \end{array}\right],B=\left[\begin{array}{c} 0\\ 0\\ \vdots \\ 0\\ 1 \end{array}\right]$$

$$C=\left[\begin{array}{ccccc} b_{0} & b_{1} & b_{2} & \ldots & b_{n-1} \end{array}\right],D=0$$

This can be proven via the transformation from a state space model to a transfer function:

$$G\left(s\right)=C{\left(sI-A\right)}^{-1}B+D$$

Using the Cayley–Hamilton Theorem [39], ${\left(sI-A\right)}^{-1}$ can be expressed as

$${\left(sI-A\right)}^{-1}=\frac{\Psi (s)}{\phi (s)}$$

where $\Psi \left(s\right)\in R^{n\times n}$ is a the adjucate matrix with entries that are polynomials in *s* of degree at most n−1.

$C{\left(sI-A\right)}^{-1}B$ can then be expressed as

$$C{\left(sI-A\right)}^{-1}B=\frac{1}{\phi (s)}\sum_{i=1}^{n}b_{i-1}\Psi_{in}\left(s\right)$$

Then, using the fact that (sI−A)Ψ(s)=ϕI, the final column of $\Psi_{in}\left(s\right)$ can be solved by linear combination to be

$$\Psi_{in}\left(s\right)=s^{i-1}$$

Therefore, the equation simplifies to

$$C{\left(sI-A\right)}^{-1}B=\frac{1}{\phi (s)}\sum_{i=1}^{n}s^{i-1}\Psi_{in}\left(s\right)=G\left(s\right)$$
